# Five percent weight loss is a significant 1-year predictor and an optimal 5-year cut-off for reducing the number of obesity-related cardiovascular disease risk components: the Japan Obesity and Metabolic Syndrome Study

**DOI:** 10.3389/fendo.2024.1343153

**Published:** 2024-03-27

**Authors:** Hajime Yamakage, Takaaki Jo, Masashi Tanaka, Sayaka Kato, Koji Hasegawa, Izuru Masuda, Munehide Matsuhisa, Kazuhiko Kotani, Mitsuhiko Noda, Noriko Satoh-Asahara

**Affiliations:** ^1^ Department of Endocrinology, Metabolism, and Hypertension Research, Clinical Research Institute, NHO Kyoto Medical Center, Kyoto, Japan; ^2^ Department of General Internal Medicine, Fushimi Momoyama General Hospital, Kyoto, Japan; ^3^ Department of Rehabilitation, Health Science University, Minamitsuru-gun, Japan; ^4^ Department of Endocrinology and Metabolism, Graduate School of Medical Science, Kyoto Prefectural University of Medicine, Kyoto, Japan; ^5^ Division of Translational Research, Clinical Research Institute, NHO Kyoto Medical Center, Kyoto, Japan; ^6^ Diabetes Therapeutics and Research Center, Institute of Advanced Medical Sciences, Tokushima University, Tokushima, Japan; ^7^ Division of Community and Family Medicine, Jichi Medical University, Shimotsuke, Japan; ^8^ Department of Diabetes, Metabolism and Endocrinology, Ichikawa Hospital, International University of Health and Welfare, Ichikawa, Japan; ^9^ Department of Endocrinology and Diabetes, Saitama Medical University, Moroyama, Japan; ^10^ Department of Metabolic Syndrome and Nutritional Science, Research Institute of Environmental Medicine, Nagoya University, Nagoya, Japan

**Keywords:** obesity, weight loss, metabolic syndrome, cohort study, 5-year follow-up

## Abstract

**Objective:**

This study aimed to identify the amount of weight loss needed in patients with obesity to improve metabolic syndrome (MetS), a risk factor for cardiovascular disease (CVD), over a long period of time.

**Methods:**

A total of 576 patients with obesity were enrolled in this study. Effects of continuous physician-supervised weight loss on the cumulative MetS components excluding abdominal circumference (defined as obesity-related CVD risk score) were investigated during a 5-year follow-up period. The extent of weight loss required to reduce the obesity-related CVD risk components was assessed using receiver operating characteristic (ROC) curve analyses.

**Results:**

Of the 576 participants, 266 completed 5-year follow-up, with 39.1% and 24.1% of them achieving ≥5.0% and ≥7.5% weight loss at the 5-year follow-up, respectively. The area under the ROC curve for reducing the obesity-related CVD risk components was 0.719 [0.662–0.777] at 1 year and 0.694 [0.613–0.775] at 5 years. The optimal cut-off value for weight loss was 5.0% (0.66 sensitivity and 0.69 specificity) and the value with 0.80 specificity was 7.5% (0.45 sensitivity) at 5 years. Greater reductions in weight were associated with greater improvements in the obesity-related CVD risk score at all follow-up periods (*P*-trend <0.001). Obesity-related CVD risk score was significantly improved by 5.0–7.5% and ≥7.5% weight loss at 1 year (*P* = 0.029 and *P* < 0.001, respectively) and ≥7.5% weight loss at 5 years (*P* = 0.034).

**Conclusions:**

A weight loss of ≥5.0% at 1 year and ≥7.5% at 5 years could reduce the number of obesity-related CVD risk components in patients with obesity.

## 1 Introduction

Obesity rates are rising globally (1), posing adverse health outcomes and contributing to developing metabolic syndrome (MetS) (2). It increases the risk of cardiovascular disease (CVD) (3). Therefore, there is a need to develop a weight management program that is effective for improving MetS and reducing the risk of CVD.

Contemporary guidelines state that a 5.0% or greater weight loss by dietary and exercise intervention is clinically important for individuals with overweight or obesity, based on epidemiological and interventional evidence (4–6). Additionally, it is also advised that individuals who are overweight or obese with MetS lose 5.0% of their body weight in order to manage this condition (7). Furthermore, a previous study reported that a 6-month lifestyle-induced weight loss program resulting in a >16% weight loss from baseline had a positive impact on MetS prevalence in Caucasian participants. This effect was observed over a 5-year follow-up period after the end of the program (8). In Japanese patients with obesity or MetS, a loss of ≥3.0% of baseline weight by a 6-month lifestyle modification program also improved obesity-related metabolic derangements at 6 months of follow-up after the end of the intervention (9). Moreover, our research group revealed that >5.0% weight loss from the baseline after 3 months of intervention beneficially influenced parameters of glycemic control, renal function, and arterial stiffness in patients with obesity in a National Hospital Organization cohort (10). These findings highlight the beneficial effects of weight loss on the management of MetS; however, the extent of weight loss from baseline to improve MetS over long periods has not been established in patients with obesity.

Because an increase in the number of MetS components elevates the risk of CVD incident (11), it is necessary to identify how much weight loss from baseline could reduce one or more MetS components in patients with obesity. Moreover, the extent of weight loss that exhibits long-term beneficial effects on the cumulative MetS components needs to be clarified, although the relationship between long-term weight loss and the cumulative number of MetS components has not been fully elucidated due to the challenging characteristics of maintaining weight loss (8, 12). To date, no prospective studies have addressed these issues in patients with obesity.

In the present study, to identify the extent of weight loss that beneficially impacts MetS in patients with obesity over long periods, we investigated the effects of a 5-year continuous physician-supervised intervention on the components of MetS excluding abdominal circumference (as obesity-related CVD risk) in outpatients with obesity in a multicenter cohort of the National Hospital Organization.

## 2 Materials and methods

### 2.1 Study design

A retrospective cohort study based on a prospective cohort study (Japan Obesity and Metabolic Syndrome Study [JOMS]) evaluated the effects of weight loss on the risk of CVD in patients with obesity in Japan.

### 2.2 Patients

The study population included patients with obesity aged between 20 and 79 years with a body mass index (BMI) of 25 or higher who visited the participating centers for their first or second visit between April 2005 and March 2007. The Japan Society for the Study of Obesity uses a BMI ≥25 for patients with obesity since this level is standardized to correspond to the international coordination of the World Health Organization (WHO) criteria for obesity, and evidence shows that obesity-related complications increase for a BMI ≥25 among the Japanese population (13, 14). In the present study, patients with obesity with BMI ≥25 with or without obesity-related health issues were included. The exclusion criteria were those with severe hepatic dysfunction and severe renal dysfunction.

A total of 576 Japanese outpatients with obesity (250 men and 326 women, mean age: 51.6 years) were consecutively enrolled in a multi-center study (JOMS), which involved five National Hospital Organization hospitals (Kyoto, Tokyo, Nagoya and Kokura Medical Centers, and Mie Hospitals) and Oishi Clinic in Japan as part of a study conducted by the Policy Based Medical Service Network for Endocrine and Metabolic Diseases during the period from October 2005 to March 2007 (15, 16).

The patients received lifestyle guidance, mainly diet and exercise therapy, for weight loss, as recommended by the Japan Atherosclerosis Society’s “Guidelines for the diagnosis and treatment of atherosclerotic cardiovascular disease” (17). The patients were instructed to consume dietary therapy consisting of 25 kcal/kg of ideal BW per day and walk at least 8000 steps per day. Since the ideal BMI for the Japanese population is considered to be 22 kg/m^2^ (17), the ideal BW was defined as 22 × the square of the subject’s height (ideal BMI [22 kg/m^2^] × height [m]^2^) in the present study. In addition, dietary and exercise guidance from a physician or nutritionist was provided at least once every three months. They are recommended to consume 60% of their total energy as carbohydrates, 20–25% as fat, and 15–20% as protein. When the patients with obesity had complications, such as type 2 diabetes, dyslipidemia, and/or hypertension, they received medications for each disease. However, they did not receive any medications for weight loss, including probiotics (18).

This study was approved by each institution’s ethical committee, and written informed consent was obtained from all patients. This study was conducted following the Declaration of Helsinki and Ethical Guidelines for Medical and Health Research Involving Human Subjects. The JOMS has been registered in the University Hospital Medical Information Network (UMIN) system (UMIN Study ID: 000000559), which is publicly available.

### 2.3 End-point definition: change in obesity-related CVD risk score

The primary endpoint was the change in obesity-related CVD risk scores between the baseline and follow-up periods. Obesity-related CVD risk score used as an endpoint in this study was the number of matches of four criteria: triglyceride (TG) level, high-density lipoprotein cholesterol (HDL-C) level, blood pressure, and fasting blood glucose level, to the exclusion of abdominal circumference from the NCEP-ATP III MetS risk score corresponding to Japanese standard cut-off values, referring to previous reports (19, 20). Cut-off values of these parameters for MetS were selected according to the guidelines of the Japan Society for the Study of Obesity (21): waist circumference ≥ 85 (men) and 90 (women) cm; fasting plasma glucose (FPG) ≥ 6.1 mmol/L; systolic blood pressure (SBP) ≥ 130 and/or diastolic blood pressure (DBP) ≥ 85 mmHg; TG ≥ 1.7 mmol/L; HDL-C < 1.0 mmol/L.

It has been reported that higher MetS risk scores, as defined by NCEP-ATP III, are associated with cumulative cardiovascular events (13, 22). However, since weight loss would reduce abdominal circumference evidently, the effects of weight loss on improving obesity-related CVD risk would be overestimated, if abdominal circumference were included in the risk score of the endpoint. Therefore, in this study, the obesity-related CVD risk score that excluded abdominal circumference from the MetS risk score was used as the endpoint.

At each follow-up period, the obesity-related CVD risk score increased by one when the patient’s value exceeded the respective standard value or when a new drug treatment was initiated. In addition, the score decreases by one when a patient who was above the criteria or on medication for each disease was below the standard value, and the medication was terminated. The obesity-related CVD risk score is expressed as a score between 0 and 4, which is the number of matches of four criteria (TG level, HDL-C level, blood pressure, and FPG level), as described above. The endpoint, the amount of change, is expressed as a value between +4 and −4. The highest improvement is −4.

### 2.4 Data collection and laboratory assay methods

At 3, 12, and 60 months after weight reduction therapy, we measured MetS-related parameters (BMI, waist circumference, SBP, and DBP) and blood parameters (FPG, hemoglobin A1c [HbA1c], TG, total cholesterol, HDL-C, and low-density lipoprotein cholesterol [LDL-C]).

BMI was calculated as weight in kilograms divided by the square of height in meters as an index of obesity. SBP and DBP were measured twice by using an automatic electronic sphygmomanometer (BP-103iII; Nippon Colin, Komaki, Japan). Blood was collected from the antecubital vein in the morning after a 12-hour fasting period without taking medication to determine FPG, HbA1c, TG, total cholesterol, HDL-C, and LDL-C, according to standard procedures (15).

### 2.5 Statistical analysis

To detect a one-point obesity-related CVD risk score difference (SD = 2 points) between the weight loss and non-loss groups, 64 cases per group were needed at a 5.0% significance level and 80% power. To obtain 64 patients from each weight loss group, a total of 384 patients were required. Considering the variability in the number of participants and the occurrence of dropouts, a sample size of 580 cases was used.

The clinical characteristics of the patients at baseline were expressed as mean and standard deviation (SD), standard error (SE), or median and interquartile range. Categorical variables were expressed as headcounts and percentages.

For the time-series trend of weight loss, the weight change rate was divided into six groups, and the percentages were expressed as bar graphs for each follow-up period.

Receiver operating characteristic (ROC) analysis was used to determine the discriminative ability of the weight reduction rate to reduce at least one obesity-related CVD risk score and the cut-off value for the weight reduction rate. Discriminatory ability was evaluated by calculating the area under the curve (AUC). For each follow-up period, the optimal cut-off value was calculated using the Youden index method and a cut-off value that would ensure a specificity of 80% to more reliably reduce the obesity-related CVD risk score.

The association between the rate of weight change and the amount of change in the obesity-related CVD risk score and the mean difference in the obesity-related CVD risk score between the baseline and follow-up periods were shown in the six groups according to the rate of weight loss. One-way Analysis of Covariance (ANCOVA)-based trend tests and paired comparisons (within ±1% pairwise weight loss) with age and sex as covariates were performed.

In the sensitivity analysis, only 547 metabolically unhealthy patients with obesity, excluding 29 metabolically healthy individuals with obesity without diabetes, dyslipidemia, or hypertension, were analyzed in the same manner as the primary endpoint. The changes in MetS risk score (which had abdominal circumference in addition to four obesity-related CVD risk score components described above) were also analyzed in the total population (n = 576).

All statistical analyses were performed using SPSS Statistics ver. 24.0 (IBM Japan, Ltd., Tokyo, Japan) and *P* < 0.05 was defined as statistically significant.

## 3 Results

### 3.1 Baseline clinical characteristics

The baseline patient characteristics are summarized in **Table 1**. The mean age was 51.6 ± 14.0 years, and 326 (56.6%) patients were women. The mean BMI was 31.4 ± 5.9 kg/m^2^ and 116 (20.1%) patients had a BMI ≥35. A total of 570 (99.0%) patients had at least one MetS component, and 441 (76.6%) had MetS. Approximately 40% of the participants were receiving medications for each MetS component (diabetes, 36.6%; dyslipidemia, 44.1%; hypertension, 45.3%). A total of 547 (95.0%) patients had at least one obesity-related CVD risk component.

**Table 1:** Baseline characteristics of patients with obesity.

	Total		
Sex (male/female) [n]	250	/	326
Age (years) [mean ± SD]	51.6	±	14.0
Body weight (kg)	82.3	±	19.6
BMI (kg/m^2^)	31.4	±	5.9
≥ 35kg/m^2^ (n, %)	119	,	20.7
Waist circumference (cm)	101.5	±	13.4
SBP (mmHg)	140.5	±	18.7
DBP (mmHg)	83.9	±	12.0
FPG (mmol/L)	7.0	±	2.8
HbA1c (%)	6.4	±	1.4
HbA1c (mmol/mol)	46.4	±	15.3
Total cholesterol (mmol/L)	5.4	±	1.4
TG (mmol/L)	1.6	[1.2, 2.4]	
HDL-C (mmol/L)	1.4	±	0.4
LDL-C (mmol/L)	3.3	±	0.8
Complications (n, %)			
Diabetes	256	,	44.4
Dyslipidemia	441	,	76.6
Hypertension	380	,	66.0
Medication (n, %)			
for diabetes	211	,	36.6
for dyslipidemia	254	,	44.1
for hypertension	261	,	45.3
MetS components (n, %)			
Waist circumference ≥ 85/90 cm	516	,	89.6
FPG ≥ 6.1 mmol/L	329	,	57.1
SBP ≥ 130 and/or DBP ≥ 85 mmHg	477	,	82.8
TG ≥ 1.7 mmol/L	376	,	65.3
HDL-C < 1.0 mmol/L	287	,	49.8
MetS risk score (n, %)			
0	6	,	1.0
1	38	,	6.6
2	91	,	15.8
3	144	,	25.0
4	145	,	25.2
5	152	,	26.4
Obesity-related CVD risk score (n, %)			
0	29	,	5.0
1	91	,	15.8
2	145	,	25.2
3	152	,	26.4
4	159	,	27.6

Data are expressed as mean ± standard deviation (SD), median [interquartile range], or the number and percentage of patients. BMI, Body mass index; SBP, systolic blood pressure; DBP, diastolic blood pressure; FPG, fasting plasma glucose; HbA1c, hemoglobin A1c; TG, triglycerides; HDL-C, high-density lipoprotein cholesterol; LDL-C, low-density lipoprotein cholesterol; MetS, Metabolic syndrome.

**Supplementary Figure 1** presents a flowchart of the study. Of the 576 participants, 168 dropped out at 12 months, and 142 dropped out at 60 months. A total of 266 (47.9%) patients were followed up until the end of the study. The reasons for patients’ dropout included changes in the living environment (91 cases), transfer to other clinics due to successful weight loss (89 cases), retirement of the attending physician (65 cases), self-interruption for unknown reasons (33 cases), and the occurrence of death or cardiovascular events (31 cases).

### 3.2 Time series of weight loss

**Figure 1:**
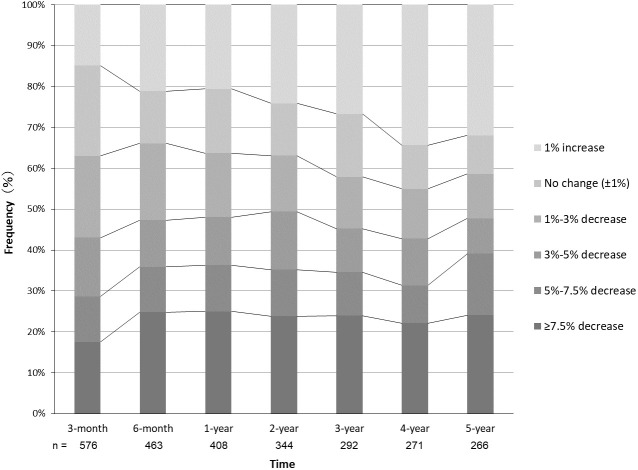
Time series of physician-supervised weight loss in patients with obesity. Frequencies of patients with the respective changes of weight from baseline are shown at each follow-up period.

The time series of weight loss from the baseline are shown in **Figure 1**. In the 3^rd^ month of the study, 43.1% of the patients achieved a weight loss of ≥3.0%, 28.6% achieved ≥5.0%, and 17.5% achieved ≥7.5%. The average weight loss was –3.3 ± 5.2%. At the 1-year follow-up, 48.0% of the patients had achieved a weight loss of ≥3.0%, 36.3% had achieved ≥5.0%, and 25.0% had achieved ≥7.5%. The average weight loss was –4.3 ± 7.4%. At 5 years of follow-up, 47.7% of the patients achieved a weight loss of ≥3.0%, 39.1% achieved ≥5.0%, and 24.1% achieved ≥7.5%. The average weight loss was –3.0 ± 8.8%.

### 3.3 Weight loss that is expected to reduce obesity-related CVD risk score by one or more

**Figure 2:**
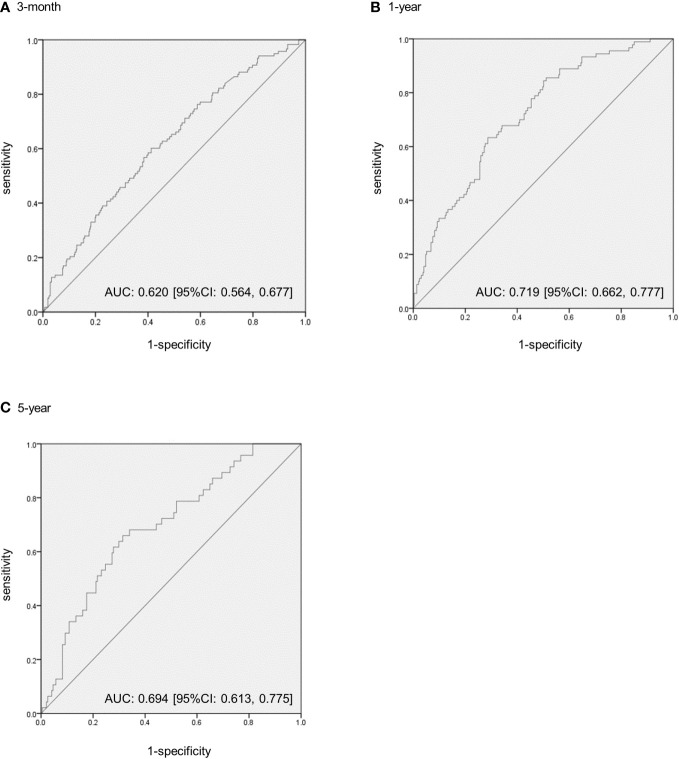
The receiver operating characteristic (ROC) curves to predict the extent of weight loss from baseline to reduce one or more obesity-related cardiovascular disease risk components in patients with obesity. **(A)** The ROC–area under the curve (AUC) at 3 months. **(B)** The ROC–AUC at 1 year. **(C)** The ROC–AUC at 5 years. CI, confidence intervals.

At 3 months, 1 year, and 5 years, 118 (20.5% [118/576]), 90 (22.1% [90/408]), and 47 (17.7% [47/266]) cases had reduced at least one obesity-related CVD risk component, respectively. **Figure 2** shows the ROC curves for the percentage weight loss from baseline for the reduction of one or more obesity-related CVD risk components. The ROC–AUC of weight loss for a reduction in one or more obesity-related CVD risk components at 3 months was 0.620 [95% confidence interval: 0.564–0.677] (**Figure 2**). The ROC–AUC at 1 year was 0.719 [0.662, 0.777], and that at 5 years was 0.694 [0.613, 0.775] (**Figures 2B, C**). The optimal cut-off values for weight loss using the Youden Index were 2.7% at 3 months (sensitivity 0.60, specificity 0.59), 5.0% at 1 year (sensitivity 0.63, specificity 0.71), and 5.0% at 5 years (sensitivity 0.66, specificity 0.69). Furthermore, the weight loss cut-off values that could ensure a specificity of 0.80 or higher for a more reliable weight loss effect were 5.8% at 3 months (sensitivity 0.35, specificity 0.80), 7.5% at 1 year (sensitivity 0.43, specificity 0.80), and 7.5% at 5 years (sensitivity 0.45, specificity 0.80).

### 3.4 Change in obesity-related CVD risk score by weight loss

**Figure 3:**
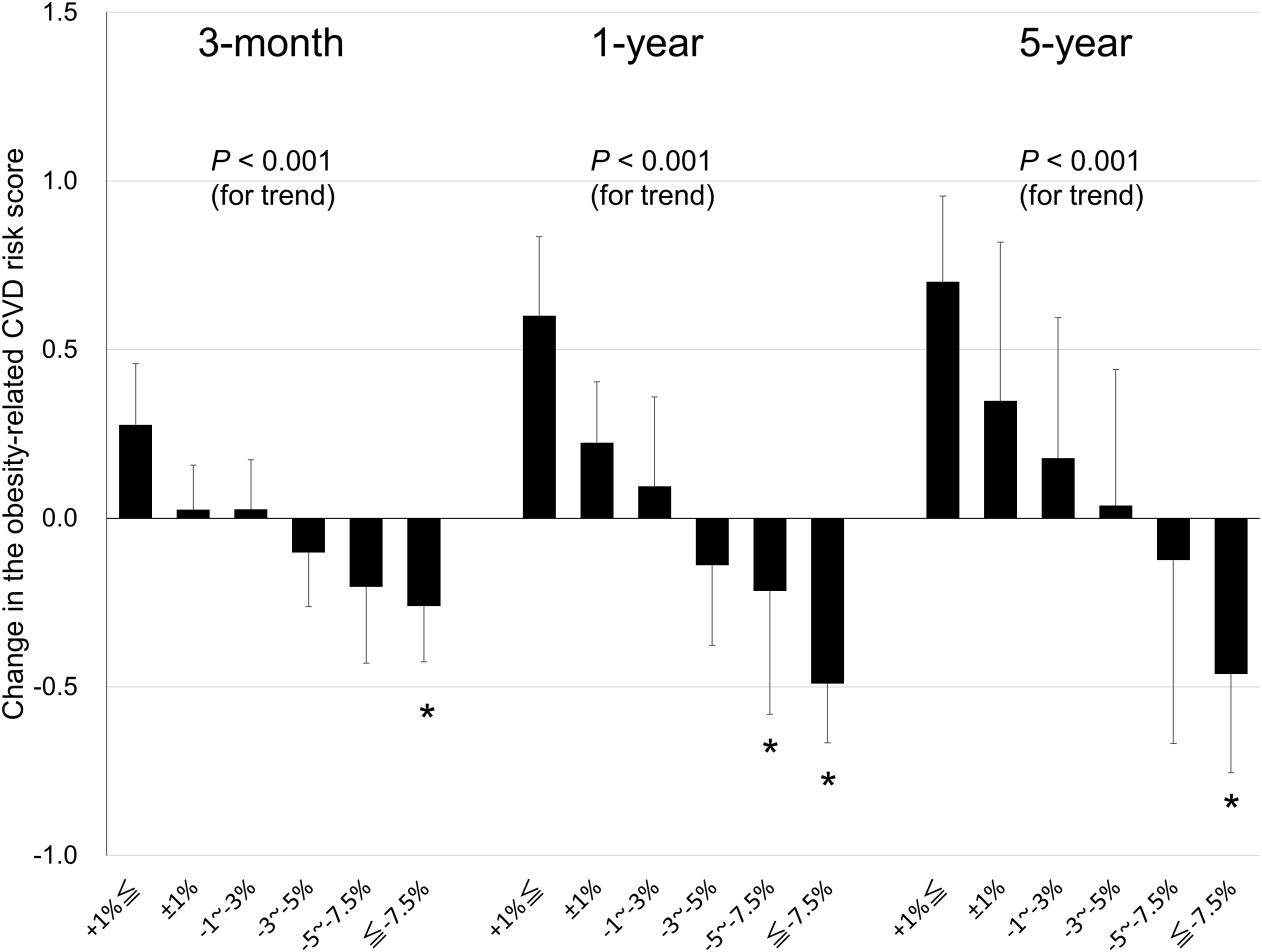
Relationship between weight loss and changes in obesity-related cardiovascular disease (CVD) risk score in patients with obesity at the 3-month, 1-year, and 5-year follow-up. Data are expressed as mean ± standard error. *P < 0.05 for the reference group (a weight change of ±1%) vs. the other group.

The relationship between weight loss and the change in the obesity-related CVD risk score is shown in **Figure 3**, which was the primary endpoint. During each follow-up period, the obesity-related CVD risk score decreased significantly in the higher weight loss group (linear trend test, *P* < 0.001). Compared to the group with a weight change of ±1% (reference group), significant improvement was observed in the group that achieved a weight loss of ≥7.5% at 3 months (mean difference of obesity-related CVD risk score: 0.03 [SE = 0.13] vs. –0.26 [0.16]; *P* = 0.009), 5.0–7.5% and ≥7.5% at 1 year (0.22 [0.18] vs. –0.22 [0.37], *P* = 0.029 in 5.0–7.5%; –0.49 [0.18], *P* < 0.001 in ≥7.5%), and ≥7.5% at 5 years (0.35 [0.47] vs. –0.46 [0.29]; *P* = 0.034).

The same sensitivity analysis was performed only for metabolically unhealthy patients with obesity at baseline (n = 519) (**Supplementary Figure 2**). At each follow-up period, the higher the weight loss, the more significantly the obesity-related CVD risk score was reduced (linear trend test, *P* < 0.001). Compared to the group with a weight change of ±1% (reference group), significant improvement was observed in the group that achieved a weight loss of ≥7.5% at 3 months (mean difference of obesity-related CVD risk score: 0.01 [0.13] vs. –0.27 [0.17]; *P* = 0.017), 5.0–7.5% and ≥7.5% at 1 year (0.20 [0.18] vs. –0.22 [0.37], *P* =0.039 in 5.0–7.5%; –0.51 [0.18], *P* < 0.001 in ≥7.5%), and ≥7.5% at 5 years (0.22 [0.47] vs. –0.41 [0.30]; *P* = 0.046). Similar results were obtained for the sensitivity analysis.

A further sensitivity analysis was performed using the MetS risk score, which included abdominal circumference as a component, as the endpoint (n = 576) (**Supplementary Figure 3**). At each follow-up period, the higher the weight loss, the more significantly the MetS risk score was reduced (linear trend test, *P* < 0.001). Compared to the group with a weight change of ±1% (reference group), significant improvement was observed in the group that achieved a weight loss of ≥7.5% at 3 months (mean difference of MetS risk score: 0.05 [0.14] vs. –0.53 [0.21]; *P* = 0.002), 5.0–7.5% and ≥7.5% at 1 year (0.29 [0.21] vs. –0.49 [0.37], *P* = 0.019 in 5.0–7.5%; –0.76 [0.20], *P* < 0.001 in ≥7.5%), and ≥7.5% at 5 years (0.35 [0.51] vs. –0.75 [0.34]; *P* = 0.022). Because of the inclusion of abdominal circumference as an endpoint, the score improvement due to weight loss was more pronounced than that of the obesity-related CVD risk score.

### 3.5 Obesity-related CVD risk components that improve with weight loss treatment

Of the 408 patients who completed the 1-year follow-up, 92 (22.5%) increased and 88 (21.6%) reduced one or more components of obesity-related CVD risk, respectively, and 228 (55.9%) had no change in the obesity-related CVD risk components.

Of the 141 patients who achieved at least 5.0% weight loss in 1 year, 57 (40.4%) had a reduction in one or more components of obesity-related CVD risk. According to the obesity-related CVD risk components, there were 24 (17.0%) patients with blood pressure improvement, 22 (15.6%) FPG improvement, 17 (12.1%) TG improvement, and 16 (11.0%) HDL-C improvement. Of the 96 patients who achieved at least 7.5% weight loss in 1 year, 41 (42.7%) showed a reduction in one or more components of obesity-related CVD risk. There were 17 (17.7%) cases of blood pressure improvement, 17 (17.7%) FPG improvement, 12 (12.5%) TG improvement, and 11 (11.5%) HDL-C improvement.

Of the 266 patients who completed the 5-year follow-up, 80 (30.1%) increased and 47 (17.7%) reduced at least one obesity-related CVD risk component, respectively, and 139 (52.3%) had no change in the obesity-related CVD risk components.

At 5 years of follow-up, of the 92 patients who achieved at least 5.0% weight loss, 31 (33.7%) had a reduction in one or more components of obesity-related CVD risk. There were 10 (10.9%) patients with blood pressure improvement, 11 (12.0%) FPG improvement, 14 (15.2%) TG improvement, and 13 (14.1%) HDL-C improvement. Of the 59 patients who achieved at least 7.5% weight loss in 5 years, 22 (37.3%) showed a reduction in one or more obesity-related CVD risk components. There were 6 (10.2%) patients with blood pressure improvement, 8 (13.6%) FPG improvement, 10 (16.9%) TG improvement, and 9 (15.3%) HDL-C improvement.

## 4 Discussion

The present study showed that the optimal cut-off value for weight loss was 5.0% to reduce one or more components of obesity-related CVD risk in patients with obesity for up to 5 years. Moreover, there was a need to achieve a weight loss of 7.5% from the baseline for these patients to reduce the number of obesity-related CVD risk components at 1 and 5 years, considering a cut-off value with a specificity of 0.80. These findings provide novel insights into the extent of weight loss in weight management programs aimed at improving the MetS components, particularly those excluding abdominal circumference, in patients with obesity.

We found the cut-off value for weight loss to be 5.0% with optimal sensitivity and specificity, and 7.5% with a specificity of 0.80, which is needed in patients with obesity to reduce the number of obesity-related CVD risk components for up to 5 years. Moreover, greater decreases in weight were significantly associated with greater improvements in the obesity-related CVD risk score in patients with obesity, irrespective of the concurrent presence or absence of MetS, at the 3-month, 1-year, and 5-year follow-ups. Conversely, only a loss of ≥7.5% weight from baseline among all the weight loss-stratified groups significantly reduced the obesity-related CVD risk score when compared with the reference group at all the follow-up periods in both patient groups. Accordingly, these findings highlight the possibility that weight loss of ≥7.5%, at least ≥5.0%, would be desirable for patients with obesity to improve obesity-related CVD risk components in the first 3 months to 5 years. In this context, a previous study reported that weight loss within the first two months predicted long-term weight loss for up to 8 years (23), further suggesting the significance of optimizing the outcome during early intervention periods to exhibit long-term beneficial effects (8). Moreover, obesity exacerbates MetS (24), thereby highlighting the need to achieve weight loss to counterbalance the detrimental effects of obesity. Thus, the effective extent of weight loss would be 7.5% or more to improve the obesity-related CVD risk components in patients with obesity based on the early improvement of the obesity-related CVD risk score, the cut-off value for weight loss, and the long-term beneficial effects on the obesity-related CVD risk score that were elucidated in this study.

Long-term maintenance of weight loss has been challenging because weight is typically lost rapidly by intervention but followed by progressive regain (8, 12); it is reported that ≥90% of individuals regained some of the weight after weight loss (25, 26). In the present study, outpatients with obesity continuously underwent physician-supervised intervention for up to 5 years, and 47.7%, 39.1%, and 24.1% of these patients achieved ≥3.0%, ≥5.0%, and ≥7.5% weight loss from baseline at the 5-year follow-up, respectively. Therefore, the results of this study revealed the effects of weight loss on the components of MetS in patients with obesity more appropriately than those of annual health checkups. In this context, the extent of weight loss for beneficial effects on MetS in patients with obesity differs between the present study and previous studies (9). Reportedly, a loss of ≥3.0% of baseline weight by 6-month lifestyle modification program after health checkups improved obesity-related metabolic parameters and MetS components in Japanese patients with obesity or MetS at six months after the end of the intervention (9), thereby suggesting that weight loss of ≥3.0% would beneficially affect obesity-related metabolic derangements in these patients for up to 6 months (9). Conversely, in this study, a loss of 3.0–5.0% weight did not significantly improve MetS at all the follow-up periods; this might be due to the fact that the mean BMI differed between the present study (31.4 ± 5.9 kg/m^2^) and the previous study (27.7 ± 2.5 kg/m^2^). Another possibility is that outpatients with obesity had more serious psychological and social issues than individuals who attended health checkups, since obesity is related to these comorbidities (27). Nevertheless, based on the findings of this study that investigated the effects of 5-year continuous physician-supervised intervention, ≥7.5% would be the preferable extent of weight loss in light of long-term beneficial effects on components of MetS in Japanese patients with obesity.

Our findings further suggest the clinical significance of ≥5.0% weight loss to reduce the obesity-related CVD risk score in patients with obesity. Although a loss of ≥7.5% weight exhibited beneficial effects on obesity-related CVD risk score over a 5-year follow-up period, ≥7.5% weight loss might be a high-hurdle setting as realistic weight goals due to challenging characteristics of maintaining long-term weight loss (8, 28). Conversely, the optimal cut-off value for weight loss to reduce obesity-related CVD risk score components was 5.0% at 1 and 5 years, and a loss of ≥5.0% weight was a significant predictor of improving obesity-related CVD risk components at 1 year. Therefore, ≥5.0% weight loss might be effective as an initial goal in terms of patient adherence to weight loss; this could further help achieve early improvement of obesity-related CVD risk components.

Treatment responses are heterogeneous and can affect weight loss outcomes (8). However, this study shows that ≥5.0% and ≥7.5% weight loss reduced all types of MetS components, including blood pressure, FPG, TG, and HDL-C, in patients with obesity at the 1- and 5-year follow-up. MetS components were uniformly affected by weight loss, further suggesting the pleiotropic beneficial effects of weight loss on MetS components in patients with obesity. Although the detailed mechanisms remain unclear, a decrease in adipose tissue may be implicated in the weight loss-induced reduction of MetS components in these patients. The adipose tissue produces various adipokines that modulate metabolism, and the function and state of this tissue have been implicated in the pathogenesis of MetS (7, 8, 29). Therefore, the reduction in body fat mass by weight loss would result in an orchestrated improvement of MetS in patients with obesity, although the priority of the improvement may depend on the individual. Supporting these possibilities, our previous studies showed that weight loss improved adipokines such as adiponectin and leptin in patients with obesity (10, 15).

The present study used (i) MetS criteria defined by NCEP-ATPIII in 2001 (22) and (ii) cut-off values for MetS described in the guidelines of the Japan Society for the Study of Obesity in 2005 (21). However, a recent study reported that NCEP-ATPIII (waist circumference is not essential in this criteria) was better than the International Diabetes Federation criteria (waist circumference is essential in this criteria) at predicting the incidence of atherosclerotic cardiovascular diseases (30). Moreover, the cut-off values for MetS have not been changed in Japan since they were developed. Accordingly, the definition of and cut-off values for MetS in this study still remain useful as surrogate indexes of the risk of CVD, further suggesting that the findings of this study can be applied to individuals with MetS in recent years.

To prevent potential overestimation of the effects of weight loss on the improvement of obesity-related CVD risk components, abdominal circumference was excluded from the obesity-related CVD risk score in this study, because a decrease in abdominal circumference would be closely related to weight loss. Our results demonstrated the beneficial effects of weight loss on obesity-related CVD risk score, and those effects were more evident when the risk score included waist circumference. These findings corroborate the significance of weight loss in reducing the risk of obesity-related CVD in patients with obesity.

This study had some limitations. We investigated the effects of weight loss on MetS in patients with obesity without sex stratification. Sex differences may exist in the susceptibility to weight loss and subsequent effects on MetS status. The effects of potential confounding factors (e.g., smoking habits) were also not examined due to the limited sample size. Further interventional studies with larger sample sizes are required to address these issues. Another limitation is that our study included only Japanese patients; therefore, the results may not be generalizable to different races and/or ethnicities. Nevertheless, our findings will be helpful for extrapolating the target value of weight loss to improve MetS in other populations.

## 5 Conclusions

In conclusion, this study provides evidence of a novel target extent of weight loss for patients with obesity to improve the components of obesity-related CVD risk; a loss of ≥5.0% weight is a 1-year predictor of reducing the obesity-related CVD risk score and ≥7.5% weight loss would be effective for up to 5 years to improve obesity-related CVD risk components. These findings will help develop novel strategies for obesity management, focusing on the improvement of MetS, thereby contributing to reducing the risk of CVD in patients with obesity.

## Data availability statement

The original contributions presented in the study are included in the article/**Supplementary Material**. Further inquiries can be directed to the corresponding authors.

## Ethics statement

The studies involving humans were approved by the Central Ethics Committee for Clinical Research at the National Hospital Organization headquarters. The studies were conducted in accordance with the local legislation and institutional requirements. The participants provided their written informed consent to participate in this study.

## Author contributions

HY: Conceptualization, Formal analysis, Funding acquisition, Investigation, Methodology, Validation, Visualization, Writing – original draft, Writing – review & editing. TJ: Conceptualization, Investigation, Writing – original draft, Writing – review & editing. MT: Conceptualization, Funding acquisition, Writing – original draft, Writing – review & editing. SK: Writing – original draft, Writing – review & editing. KH: Writing – original draft, Writing – review & editing. IM: Writing – original draft, Writing – review & editing. MM: Writing – original draft, Writing – review & editing. KK: Writing – original draft, Writing – review & editing. MN: Writing – original draft, Writing – review & editing. NS-A: Conceptualization, Funding acquisition, Project administration, Resources, Supervision, Validation, Writing – original draft, Writing – review & editing.
