# Supplementary material for: Five percent weight loss is a significant 1-year predictor and an optimal 5-year cut-off for reducing the number of obesity-related cardiovascular disease risk components: the Japan Obesity and Metabolic Syndrome Study
Source: Front Endocrinol (Lausanne). 2024 Mar 27;15:1343153. doi: 10.3389/fendo.2024.1343153 (PMC11005029; doi:10.3389/fendo.2024.1343153)
Supplement: Supplementary file 1 [file DataSheet_1.pdf]

Supplementary Material

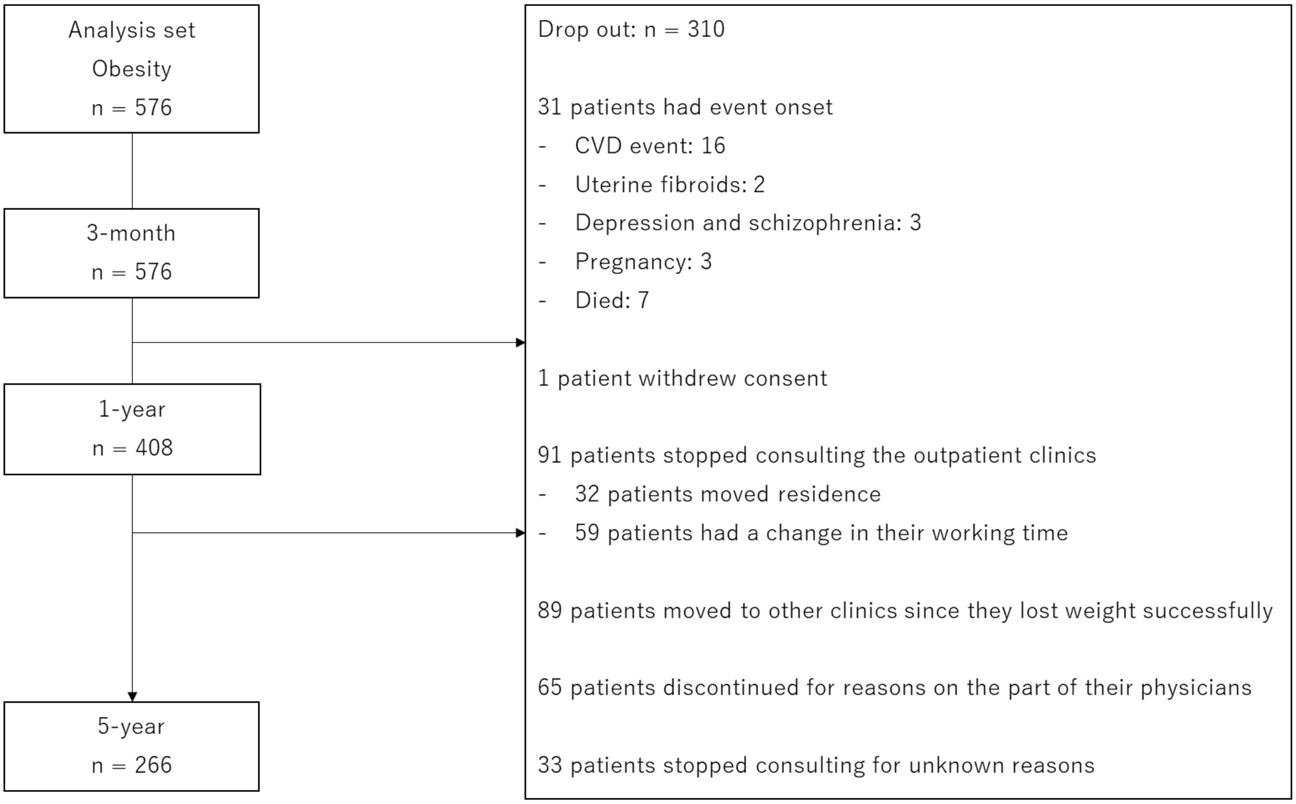

Supplementary Figure 1. Flow chart of the study. CVD, cardiovascular disease.

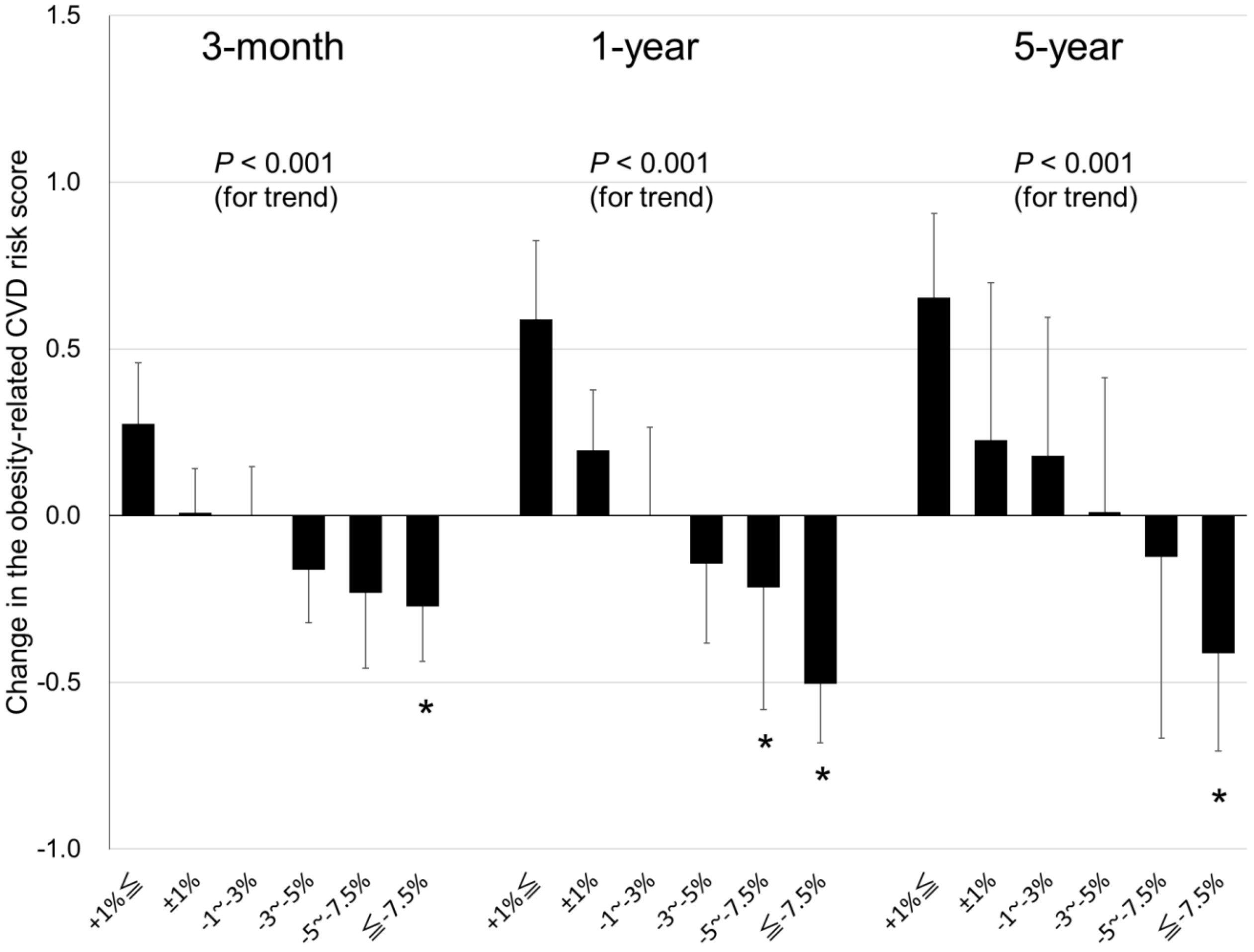

Supplementary Figure 2. Relationship between weight loss and changes in obesity-related cardiovascular disease (CVD) risk score at the 3-month, 1-year, and 5-year follow-up in metabolically unhealthy patients with obesity at baseline. Data are expressed as mean ± standard error. *$P < 0.05$ for the reference group (a weight change of ±1%) vs. the other group.

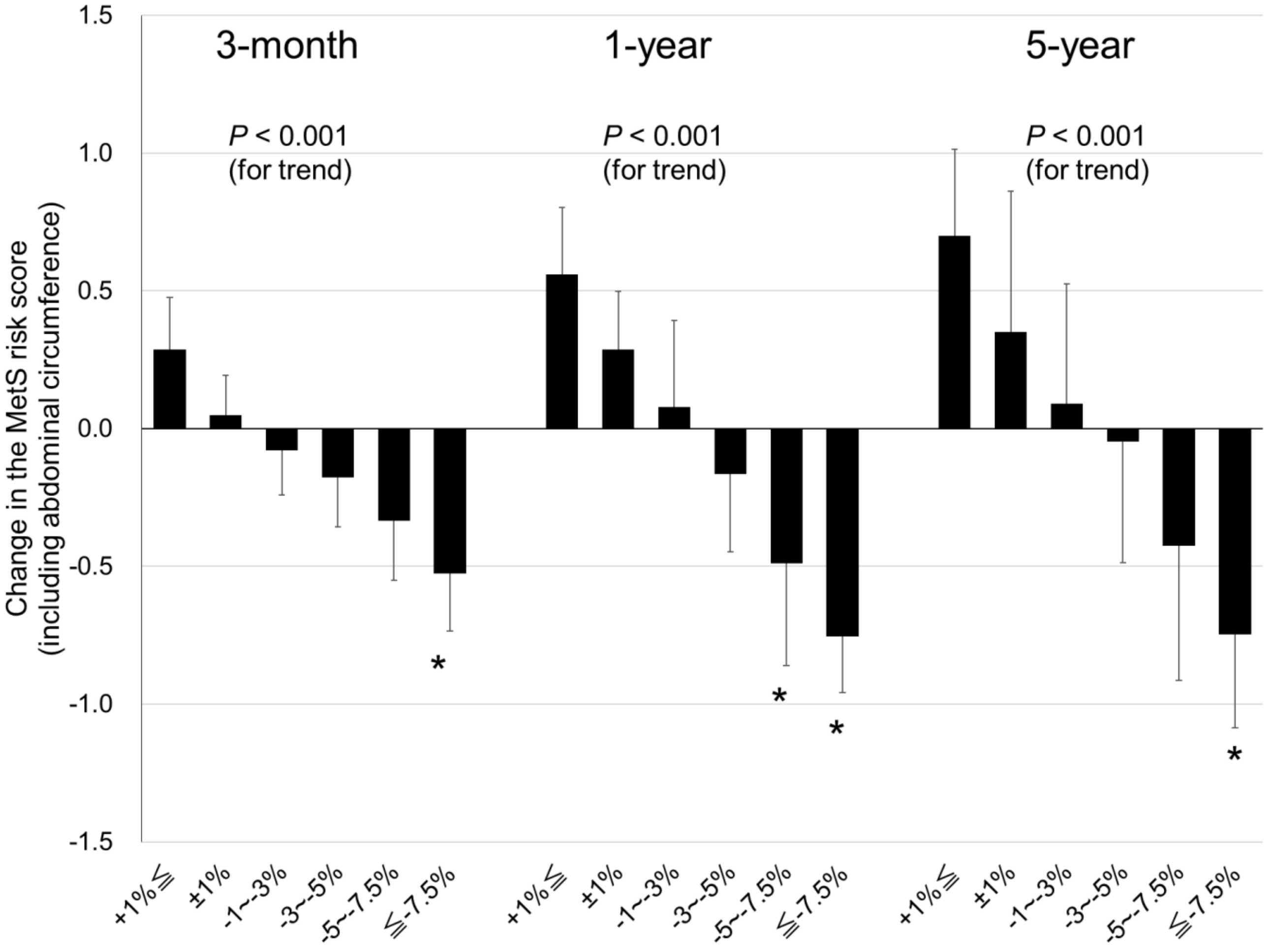

Supplementary Figure 3. Relationship between weight loss and changes in metabolic syndrome (MetS) risk score, including abdominal circumference, at the 3-month, 1-year, and 5-year follow-up in metabolically unhealthy patients with obesity at baseline. Data are expressed as mean ± standard error. *$P < 0.05$ for the reference group (a weight change of ±1%) vs. the other group.
